# Functional, Contextual, and Interventional Drivers of Formal Coercion in Acute Mental Health Units: A Feature Analysis

**DOI:** 10.1111/inm.70240

**Published:** 2026-02-24

**Authors:** Esario IV Daguman, Jacqui Yoxall, Richard Lakeman, Marie Hutchinson

**Affiliations:** ^1^ Faculty of Health Southern Cross University Coffs Harbour New South Wales Australia; ^2^ Faculty of Health Southern Cross University Lismore New South Wales Australia; ^3^ School of Nursing and Midwifery University of Southern Queensland Toowoomba Queensland Australia

**Keywords:** Boruta algorithm, coercion, mental health services, predictors, psychiatric nursing, random forests

## Abstract

Minimising formal coercion, such as seclusion, physical restraint, and forced medication, remains a global priority in acute mental health units. However, key drivers beyond individual‐level features are poorly understood. This exploratory analysis was intended to identify the top functional, contextual, and interventional features linked to formal coercion in three Australian acute adult mental health inpatient units. Nested within a mixed concurrent control study, this feature analysis examined nurses' reports of 2955 de‐escalation events over 324 days, from March 2024 to April 2025, including nurses' commentaries on the behavioural functions that triggered de‐escalations. Fifteen inductively coded functional features were analysed alongside 15 contextual and 16 interventional features. Studied target variables included seclusion and physical restraint events and their durations, as‐needed intramuscular psychotropic events, physical injury events, and Code Black activations. Features were analysed using bivariate statistics and machine learning techniques, including the Boruta algorithm for feature selection and random forest regressions for predictive modelling. Top drivers for the use of formal coercion included behavioural ‘Responses to Challenging, Physical and External Stimuli,’ incidents of self‐harm, incidents directed towards nurses, and the application of specific de‐escalation techniques. A hierarchy of behavioural functions is proposed as a by‐product of this analysis. These findings provide nuanced insights into the drivers of formal coercion and the underlying value arrangements, as well as elevate the merit of ecological, bottom‐up approaches in early warning signs work.

## 1 Introduction

Identifying clinical practices and contextual dynamics that contribute to the use of formal coercion in acute mental health units is central to improving prevention efforts. Formal coercion is the legal subset of the coercion continuum that limits a person's agency through regulated measures (Paradis‐Gagné et al. 2025). These ‘restrictive care practices’ (Belayneh et al. 2024, 1256) include events of seclusion, physical restraint, and forced medication. Despite being positioned as ‘safety measure[s] of last resort’ (RANZCP 2021, para 14), these practices are experienced as traumatising and dehumanising (Bennetts et al. 2024) and may pose a range of health risks, including physical injuries and even death (McSherry and Maker 2021). Reducing formal coercion is core to reform policy advocacy worldwide, within a context that includes localised cultures of care being seen as influential in its development and continuation (Gill et al. 2024). Context refers to ‘any feature of the circumstances in which a [reform] is conceived, developed, implemented and evaluated’ (Skivington et al. 2021, 2). Supporting this view is a multi‐centre German study that found much of the variation in formal coercion rates across 31 public psychiatric hospitals likely reflected institutional practices, even though involuntary admission rates were the strongest predictor of seclusion and restraint (Flammer et al. 2022). This variation highlights the importance of considering clinical practices, including nurses' de‐escalation interventions and functional behaviour framing, as early warning signs of pending coercion.

The functional framing of behaviour underlines the ongoing debate over the most influential reason for deploying formal coercion. Functional framing of behaviour, which professionals with expertise in understanding behaviour would call functional analysis, is the process of assessing ‘the purpose [a] behavior serves for an individual’ (Hanley et al. 2003, 148) through the identification of the contingencies from the environment that keep the behaviour going, its ‘reason.’ Aggressive behaviour has frequently been cited as a key reason, which likely explains the over 146 investigations undertaken to understand its prevalence and development (Weltens et al. 2021). Early work classifying inpatient aggression functions in forensic settings exemplified this framing, with various underlying reasons proposed, including anger expression, demand evasion, and social status assertion (Daffern et al. 2007). Others take issue with the aggressive behaviour focus, arguing that, in a 15‐year Finnish psychiatric study, agitation and disorientation were the most common reasons for seclusion and restraint, and occurred without any actual or threats of violence (Keski‐Valkama et al. 2010). A recent support for this perspective is the Dutch study of 1106 people admitted into a psychiatric hospital that found ‘disruptive behaviour and [actions considered] beneficial to [the] patient’ (Vruwink et al. 2022, 1) as nonaggressive reasons for seclusion.

A third view posits a value arrangement behind formal coercion. In a narrative review of 99 articles on ethical justifications regarding coercion, many professionals agreed that some values (e.g., freedom of movement) were less important than others (e.g., long‐term recovery and safety goals; Chieze et al. 2021). This arrangement is consistent with the hierarchy of needs by Abraham Maslow (1970), a prominent humanistic theorist, in which safety goals or needs are seen as more prepotent than the ‘desire…for independence and freedom’ (p. 45). This needs arrangement was first introduced by Maslow (1943) in his theory of human motivation, which proposes that becoming a whole person emerges from sequentially meeting needs from the physiological level (e.g., food, sex) to safety (e.g., freedom from danger), love (e.g., belonging to a family), esteem (e.g., desire for strength), and self‐actualisation (i.e., self‐fulfilment). Maslow's theory has been regarded as a practical, humanistic, and structured framework for mental health practitioners to address a person's needs and to understand the reason behind a person's behaviours (Feist et al. 2021).

A space exists for a humanistic framing of behaviour in identifying the most important reasons for the use of formal coercion, as becoming a whole human being is a vocation for many people with mental ill‐health (Anthony 2004) and people who experienced coercive engagements with mental health services (Elwyn et al. 2025; Pariseau‐Legault et al. 2019). A humanistic framing means that behaviours are not seen solely as properties of the brain, but as part of a person's whole (Moncrieff et al. 2024; Slade 2009). There is support for the importance of such framing in a survey of 95 people who received care from various mental health services in Australia, in which encounters with nurses who met their human needs were rated among the most helpful (Lakeman et al. 2022). However, people with mental ill‐health experiences have often been painted as having few strengths (Goffman 1961), with behaviours labelled as ‘dangerous’ (Large, Nielssen, et al. 2008; Large, Ryan, et al. 2008), that is, likely to cause harm to others and oneself. Behaviours labelled as dangerous have been statistically linked to an increased endorsement of formal coercion (Steiger et al. 2022), despite that such labelling devalues people's capacity to be knowers, to reason, to express themselves, and to share credible testimonies (Lakeman 2010). Averting risks of people becoming dangerous has also moved mental health professionals away from supporting people to take on challenges for growth (Felton et al. 2018). There has been no regard for behaviours being emergent products of power structures (Johnstone and Boyle 2018).

In identifying the most important reasons for formal coercion, it can be helpful to leverage machine learning (ML). This computer algorithm can be trained on complex datasets to make predictions (Iniesta et al. 2016). In aged care, ML models were instrumental in identifying cognitive impairment as a top driver of physical restraint (Wang et al. 2023). In child and adolescent psychiatric services, decision trees were used to flag seclusion and restraint risk in children, with disruptive mood dysregulation disorder and/or attention‐deficit hyperactivity disorder diagnosis as among the strongest drivers (Magnowski et al. 2022). In a forensic mental health service, various ML models helped identify past violence as a key driver of restraint, seclusion, and involuntary medication (Günther et al. 2020). In a public non‐forensic mental health service, random forest (RF) was applied, and involuntary admission was found as the most important driver of mechanical restraint (Danielsen et al. 2019). In another ML application in a public, non‐forensic mental health service, the overall measure of a person's symptom severity, treatment response, and treatment efficacy was found to be the leading driver of formal coercion (Hotzy et al. 2018).

These ML applications represent welcome advancements in predicting the use of formal coercion. However, they have not included features relating to behavioural functions that clarify the purpose or cause of behaviours, nor a broader set of nurses' real‐time de‐escalation interventions, which are said to influence formal coercion (see Price et al. 2018). Restrictive practice durations, physical injuries, and Code Blacks (hospital emergency codes for summoning security guards to support in containing incidents posing personal safety concerns; Muir‐Cochrane et al. 2020) have also not been considered as target variables. These studies have relied on individual‐level features and are inconclusive.

To address the foregoing gaps in the academic mental health literature, the current exploratory feature analysis was aimed at developing and evaluating predictive models of formal coercion by training ML on data from 324 days of 2955 nurse‐reported de‐escalation incidents in adult inpatient units. The specific objective was twofold. First, this analysis was intended to explore nurses' commentaries on the purpose behind behaviours that triggered a de‐escalation response. Second, it was intended to investigate whether these behavioural functions, when analysed alongside contextual and interventional features, predict the use of restrictive practices, event duration, physical injury, and Code Black. An ancillary objective of this analysis was to explore whether specific values (functions) are more important than others within formal coercion through Maslow's (1943) theory of human motivation.

## 2 Methods

### 2.1 Design

This feature analysis is part of a broader research project, with a prospective, multi‐site, mixed concurrent control design. The project was aimed to examine the impact and implementation of a complex intervention, deployed across three acute mental health units in New South Wales (NSW), Australia (Daguman, Taylor, Flowers, Owen, et al. 2025). The intervention includes an explicit de‐escalation framework grounded in understanding the message behind a person's behaviour during daily interactions. The mixed concurrent control design mainly applies to the evaluation and implementation stages (Skivington et al. 2021) of the broader research project. In contrast, this feature analysis pertains to the complex intervention research core element, which focuses on further developing programme theory. A programme theory articulates the implementation context features that are anticipated to influence how the intervention works (Funnell and Rogers 2011). In this feature analysis, qualitative and quantitative data were gathered and analysed separately, but concurrently. An element of integration occurred during the operationalisation of functional features. However, the main integration was undertaken during reporting and interpretation. This feature analysis was exploratory, but was pre‐planned. The statistical and algorithmic approaches employed in this feature analysis build on numerical methods used in an earlier work (Daguman, Taylor, Flowers, Lakeman, and Hutchinson 2025b) that focused only on contextual and interventional features. Reporting followed relevant guidelines from the Transparent Reporting of a multivariable prediction model for Individual Prognosis Or Diagnosis (TRIPOD; Moons et al. 2015).

Ethical approvals were granted by human research ethics committees from a local health district research office (2023/PID00297–2023/ETH00272) and a university (2023/069). Access to administrative records was approved under a waiver of consent, as no identifiable data were collected or re‐identifiable. Nurses' completion of de‐escalation logs was considered an implied consent, with no nurse identifiers recorded. Approval was granted to report aggregate sex and diagnosis data for people who received care and were involved in de‐escalation events. Identifiable details were collected solely to support log completion and then removed by a broader research project team member not involved in the analysis. A random study code was used to replace identifiers before the data were shared with the first author, who remained blinded to participant identities.

### 2.2 Setting

Data were collected from three acute adult inpatient mental health units in regional and metropolitan NSW public hospitals. Each unit provides 24‐h multidisciplinary care. As part of the broader research project in which this feature analysis is nested, each unit adopted a consistent intervention and data collection protocol. Each unit differed in its culture of care, including the integration of lived experience practitioners and approaches to reviewing incidents of restrictive practices.

### 2.3 Data Source

All features in this analysis were prospectively sourced from nurses' de‐escalation logs. At the same time, the target variables were obtained from the routinely reviewed administrative data sources (i.e., legally mandated standardised reports on seclusion and physical restraint, incident management system [ims+] reports on physical injuries and Code Black, and electronic medication management system [eMeds] reports on as‐needed intramuscular [IM] psychotropics). The de‐escalation logs were purpose‐built for the broader research project in which this feature analysis is nested, rather than part of routine documentation. The use of these logs for this feature analysis was defined a priori. The log was based on an earlier version used in a cluster analysis, which signalled the need for measures that hold an image of a psychotherapeutic nurse in a time when mental health nursing practice is often unseen or unclear (Daguman, Taylor, Flowers, Lakeman, and Hutchinson 2025a).

Log events were aggregated by study day index, which formed the unit of analysis for this feature analysis. An aggregated unit of analysis supports the identification of missing data (Bowers et al. 2015) and the consequent application of imputation methods, given that there is no true reference value for the type of count data used in this feature analysis. Daily average values for specific features, such as the number of staff involved in de‐escalation and the level of situational aggression (rated on a six‐point scale; treated as a continuous variable since its ordered categories are assumed to approximate equal intervals), were calculated across all logged events on a daily basis. Nurse educators trained nurse participants in the use of de‐escalation logs. This training covered orientation on the meaning of each item in the log (e.g., redirection vs. change of environment), the opportunity to cross‐reference log data with their clinical nursing notes made during their shift in order to minimise recall bias, and the order in which the log is to be completed to avoid reverse causality (i.e., any target variables, such as seclusion and restraint, are to be considered as the end point events).

Missing data in the logs was identified through the number of days without a completed entry. By the end of the one‐year intervention period, 2955 de‐escalation logs were available, covering 89% of the implementation days across all participating sites. Multiple imputations using the mice package (van Buuren and Groothuis‐Oudshoorn 2011) were then applied to make the dataset equivalent to a full year. No sensitivity analysis was undertaken, as it was not pre‐planned. On the other hand, data from administrative sources are subject to integrity checks as part of standard review procedures mandated by their respective policies to ensure reliability and validity. Events and durations of seclusion and physical restraint are concurrently validated against ims+ records by respective mental health service administrators, as part of due diligence in reporting to the Australian National Seclusion and Restraint Database (AIHW 2024). Code Black and physical injury events reported in ims+ are reviewed for further action by the respective mental health service administrators (NSW Health 2020). The eMeds features oversight on digitalised medication data, which has been linked to fewer medication errors, particularly in rural NSW (NSW Health 2019). The use of routine data sources for target variables was intended to reduce bias related to performance and surveillance. A day without a formal coercion event was considered a zero‐event day, not a missing data point.

Personal and clinical features (i.e., administratively‐recorded sex and diagnosis) were excluded from this feature analysis. Sex was recorded only in binary form, and diagnosis data lacked consistent terminology and coding. About 14% of 424 unique cases (does not include events involving the same person being supported) had symptom descriptions, rather than formal diagnoses, with terms like ‘acute stress reaction’, ‘family conflict’, or ‘suicidal ideation’ (see Daguman, Yoxall, Lakeman, and Hutchinson 2025) overlapping conceptually with constructed log features in this analysis. Terms such as ‘agitated behaviour’ were also not characteristic of a particular mental ill‐health diagnosis (or were not pathognomonic). To avoid misclassification bias, symptom descriptions were not reinterpreted or revised. Excluding repeat events, most de‐escalations involved males (58%). The most common diagnoses were schizophrenia (33%), schizoaffective disorder (13%), drug‐induced psychosis (13%), and bipolar disorder (11%), with all others under 10 cases.

### 2.4 Variables

A total of 46 explanatory features were included and categorised broadly into three categories: (i) 15 functional features, which were derived through thematic coding of qualitative entries in the de‐escalation logs; (ii) 15 contextual features (i.e., shift timing, observation area, number of nurses involved, direction of incidents, and situational aggression before de‐escalation); and (iii) 16 interventional features (e.g., de‐escalation responses such as acknowledgement and validation, redirection, sensory modulation, culturally sensitive care). Nine target variables were examined as outcomes (e.g., seclusion, Code Black, physical injury). The complete list of event codes, their categories, data types, and event code descriptions is presented in Table 1. Full descriptions of the behavioural function codes are provided in the Results section. At the same time, the details of the interventional features are available in the Supporting Information.

**TABLE 1:** List of codes for features and target variables, feature category, and code description.

Feature code	Feature category	Description of feature code
T01	FF	Aligning with internal reality^a^
T02	FF	Attaining emotional and physiological balance or comfort^a^
T03	FF	Reclaiming autonomy or personal space^a^
T04	FF	Seeking validation, connection, and social activity^a^
T05	FF	Expressing discontent or frustration with injustice and interference^a^
T06	FF	Mirroring another person's anger^a^
T07	FF	Establishing control or communicating strength^a^
T08	FF	Responding to uncertainty and fear^a^
T09	FF	Responding to challenging physical external stimuli^a^
T10	FF	Misinterpreting intent^a^
T11	FF	Accessing valued possessions^a^
T12	FF	History of aggression^a^
T13	FF	Responding to medical intervention^a^
T14	FF	Sexually inappropriate conduct^a^
T15	FF	Witnessing other people's distress, hardship, or vulnerability^a^
AM	CF	Incident within the morning shift^a^
PM	CF	Incident within the afternoon shift^a^
Night	CF	Incident within the night shift^a^
HOA	CF	Incident within a high observation area^a^
LOA	CF	Incident within a low observation area^a^
NOS	CF	Number of nurses involved in de‐escalation^b^
DCA	CF	Agitation only, or the person was in a calm state^a^
DAP	CF	Towards another person receiving care^a^
DSH	CF	Self‐harm^a^
DPS	CF	Towards nurses or unit staff^a^
DPV	CF	Towards a visitor, by the person^a^
DVP	CF	Towards the person, by a visitor^a^
DVS	CF	Towards the nurse or unit staff, by a visitor^a^
DPH	CF	Towards properties or inanimate objects^a^
SS1	IF	Staff emotional self‐regulation or self‐management^a^
SS2	IF	Identify the issue or problem^a^
SS3	IF	Acknowledge and validate^a^
SS4	IF	Work towards an agreeable solution^a^
SS5	IF	Establish expected behaviour^a^
IDI	IF	Distraction^a^
IRE	IF	Redirection^a^
ICE	IF	Change environment^a^
IRS	IF	Reduced stimulus^a^
IMU	IF	Music^a^
IOP	IF	Oral pro re nata (PRN; as needed) medication^a^
IIN	IF	Individualised staff time^a^
IFD	IF	Food and drinks^a^
ICS	IF	Culturally sensitive care^a^
ISM	IF	Sensory modulation^a^
IPC	IF	Phone call^a^
PRD	CF	Situational aggression before de‐escalation^b^
SEC	TV	Seclusion, defined as ‘when a person is placed alone in a room and cannot leave by themselves’ (AIHW 2024, 1)^a^
RES	TV	Physical restraint, defined as ‘when staff use their hands or body to stop a person moving freely’ (AIHW 2024, 1)^a^
SED	TV	As‐needed intramuscular (IM) psychotropics includes administrations of lorazepam, midazolam, droperidol, haloperidol, olanzapine, ziprasidone, and zuclopenthixol acetate (see Fitzgerald 1999), which, regardless of the underlying rationale, can be restrictive^a^
RPC	TV	Total restrictive practice counts (sum of seclusion, physical restraint, and As‐needed IM psychotropics events)^a^
SECD	TV	Seclusion duration (in min)^b^
RESD	TV	Physical restraint duration (in min)^b^
RPD	TV	Total restrictive practice duration (sum of seclusion and physical restraint duration in minutes)^b^
CDB	TV	Code Black (emergency code that calls for security personnel's support in responding to personal safety threats)^a^
TPI	TV	Total physical injuries (sum of injuries incurred by the patient, staff and visitor; not inclusive of self‐harm)^a^

Abbreviations: CF, contextual features; FF, functional features; IF, interventional features; TV, target variables.

^a^ Count data.

^b^ Continuous outcome.

### 2.5 Data Analysis

**FIGURE 1:**
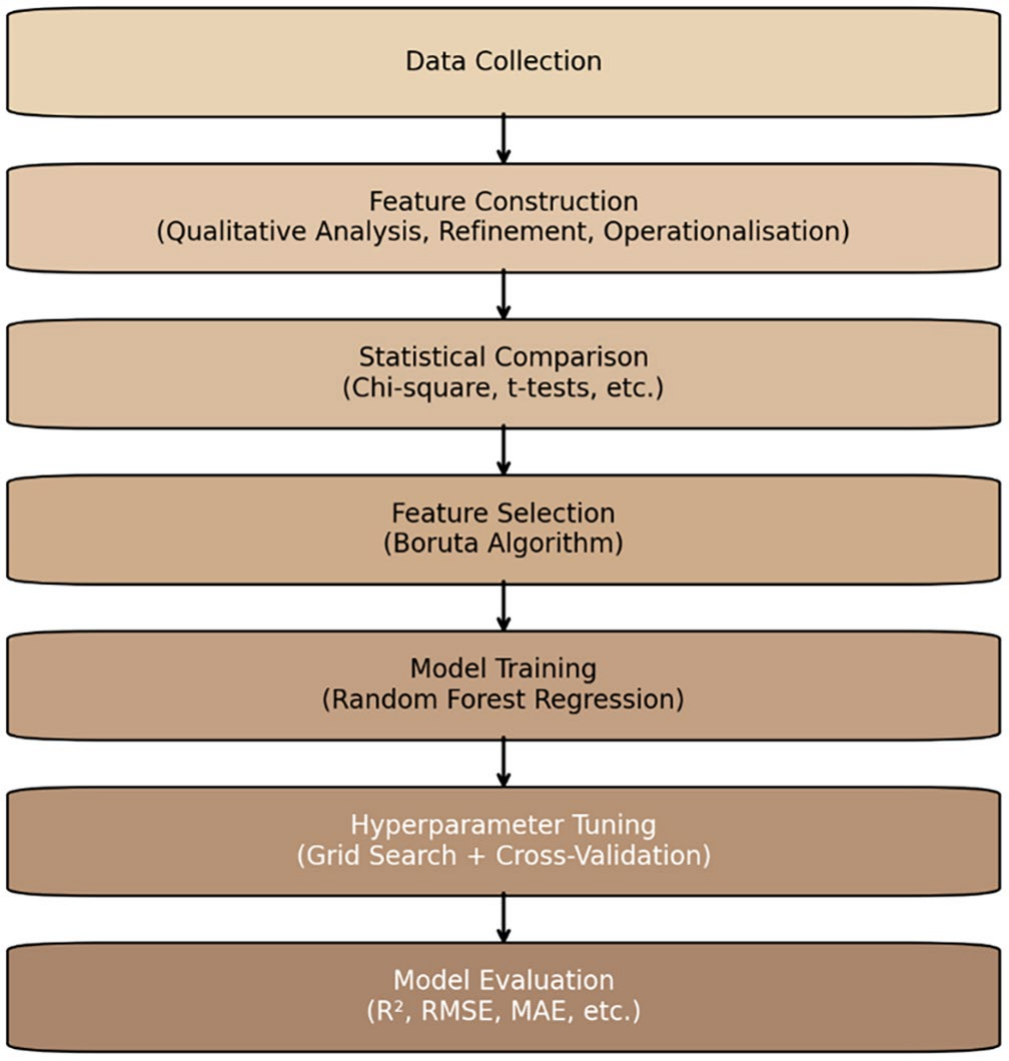
Process flow of study procedures.

Qualitative and quantitative data treatments were applied from feature construction to model evaluation. An overview of each step in the process is provided in Figure 1. All analyses were undertaken using NVivo (Lumivero 2017), R (R Core Team 2024), and RStudio (Posit Team 2023), with visualisations produced using Matplotlib (Hunter 2007).

Qualitative entries from the logs were subjected to thematic auto‐coding to support the modelling of underlying behavioural functions. Initial codes were generated using automated text search and pattern‐based coding functions in NVivo (Lumivero 2017). These were inductively refined by, and served as a reference for, the first author, who undertook a reflexive thematic analysis (Braun and Clarke 2006, 2023). This analysis was in five recursive stages: (a) re‐engaging with the data, (b) organising, (c) coding, (d) reorganising codes, and (e) developing and refining themes. First, commentaries on triggers of de‐escalation were re‐read for data familiarisation. Second, the data were organised into an initial order using a spreadsheet. Third, initial codes were developed, ensuring they reflected the purpose behind a person's behaviour. Fourth, these codes were revised and regrouped into broader categories that encompassed recurrent and prominent facets of purposeful behaviours as described by the nurse participants. Finally, these categories were further aggregated and reduced into overarching themes. Another author (MH) reviewed a coding sample of raw data and agreed with the first author, who completed the remainder of the coding. This review was aimed at enhancing interpretive breadth, rather than obtaining formal inter‐rater reliability (Åsbø et al. 2024). The number of codes per functional feature was reported to reflect the breadth of the identified themes. In contrast, the importance of each theme was determined by the researchers' interpretive judgement, rather than frequency alone. Final thematic codes were operationalised as explanatory features and mapped to Maslow's (1943) needs framework to answer this paper's ancillary objective. Given the impact of language on mental ill‐health stigma, the functional features were worded with an intention of softening the blow of any diagnostic or traumatic reality.

Statistical comparisons between days with and without restrictive practice events, Code Black, and physical injuries were performed using Chi‐square or Fisher's exact tests for categorical features, and independent *t*‐tests or Mann–Whitney‐Wilcoxon tests for continuous features, depending on the presence of expected zero values and normality assessed via Shapiro–Wilk tests, respectively. Continuous target variables (i.e., restrictive practice duration) were not analysed statistically, which would require covariate adjustment and model specification. This layer of analysis would undermine the aim of examining relationships between variables in isolation and introduce additional complexity that is unsuited to this phase of the data treatment process.

Feature selection was performed using the Boruta algorithm (Kursa and Rudnicki 2020). It identifies important predictors by comparing them to permuted shadow features within an RF framework. Confirmed features were then used to train separate RF regression models for each outcome, using the randomForest package (Breiman et al. 2022). Given the use of daily event aggregates and the count‐based nature of the outcomes, regression models were preferred over classification models to preserve variability in event frequency. Hyperparameters (ntree and mtry) were tuned via a 10‐fold cross‐validation grid search. Models were evaluated on a 20% hold‐out test set using coefficient of determination (*R* ^2^), root mean square error (RMSE), mean squared error (MSE), and mean absolute error (MAE). Feature importance was determined using the %IncMSE metric, which quantifies the effect of permuting each predictor on model error. Model performance, including tuning parameters, is summarised in Table 2.

**TABLE 2:** Random forest regression model specification and model performance before and after hyperparameter tuning.

Metric	SEC	RES	SED	RPC	SECD	RESD	RPD	CDB	TPI
Model specification	SEC ~ DPS + SS3 + ICS + SS2 + SS4 + SS1 + IIN + PM + IRE + HOA + DSH	RES ~ IRS + SS4 + DSH + SS2 + SS5 + T13 + DPS + IIN + PM	SED ~ IIN + IMU + SS2 + T13 + IOP + T03	RPC ~ ICE + DSH + IRE + DPS + SS2 + IMU	SECD ~ SS2 + ICS + IIN + SS1 + SS4 + SS3 + ICE + PM + DSH	RESD ~ DPS + SS2 + SS3 + SS4 + DSH + SS1 + SS5 + HOA + LOA + PM + IRS	RPD ~ DSH + SS1 + SS3	CDB ~ SS4 + SS3 + SS5 + IRS + DSH	TPI ~ HOA + SS2 + SS3 + SS5 + IRS + T3 + IDI + SS4 + DPS + ICS + SS1 + DSH + IRE
Baseline *R* ^2^	0.0206	0.0752	0.0071	0.0027	0.0064	0.0001	0.0113	0.0168	0.0062
Baseline RMSE	0.3334	0.5593	2.1454	2.8462	295.2809	3.4732	309.4559	0.7919	0.1763
Baseline MSE	0.1112	0.3128	4.6028	8.1006	87 190.8126	12.0630	95 762.9824	0.6272	0.0311
Baseline MAE	0.2351	0.4301	1.7700	2.2469	132.8570	1.6703	124.2239	0.5176	0.0599
Baseline parameters	ntree = 500, mtry = 3	ntree = 500, mtry = 3	ntree = 500, mtry = 2	ntree = 500, mtry = 2	ntree = 500, mtry = 3	ntree = 500, mtry = 3	ntree = 500, mtry = 1	ntree = 500, mtry = 2	ntree = 500, mtry = 3
Tuned *R* ^2^	0.0152	0.0423	0.0024	0.0006	0.0083	0.0003	0.0102	0.0353	0.0063
Tuned RMSE	0.3146	0.5577	2.1127	2.8212	281.7571	3.4604	311.2559	0.7749	0.1734
Tuned MSE	0.0989	0.3110	4.4636	7.9594	79 387.0571	11.9744	96 880.2159	0.6004	0.0301
Tuned MAE	0.2243	0.4285	1.7429	2.2315	120.5699	1.6174	123.1427	0.5104	0.0591
Tuned parameters	ntree = 800, mtry = 1	ntree = 400, mtry = 1	ntree = 200, mtry = 1	ntree = 100, mtry = 1	ntree = 100, mtry = 1	ntree = 300, mtry = 1	ntree = 100, mtry = 1	ntree = 400, mtry = 1	ntree = 700, mtry = 1

*Note:* Refer to Table 1 for feature and target variable codes, feature category, and code description. The tilde symbol (~) in a model represents that the variables on the left of the symbol are being modelled as a function of the variable(s) on the right of the symbol. For example, the value of total restrictive practice duration (RPD) is predicted by the value of self‐harm (DSH), staff emotional self‐regulation or self‐management (SS1), and acknowledgement and validation (SS3).

Abbreviations: MAE, mean absolute error; MSE, mean squared error; *R* ^2^, coefficient of determination; RMSE, root mean square error.

Multicollinearity in this feature analysis dataset was assessed through variance inflation factors. However, no features were removed, as the authors considered each represented a distinct functional, contextual, and interventional concept. Removing multicollinear features could also occlude practically meaningful differences relevant to real‐world acute inpatient care. RF is also robust to multicollinearity among features and does not reduce overall model performance (Matsuki et al. 2016; Wies et al. 2023).

## 3 Results

This study's findings are presented in two parts. The first part outlines the qualitative themes on the perceived function behind individuals' behaviours that triggered nurses' de‐escalation. These themes were considered features in the second part, which reports the quantitative analysis examining their associations with the target variables.

### 3.1 Part One: Feature Construction Outcomes

**FIGURE 2:**
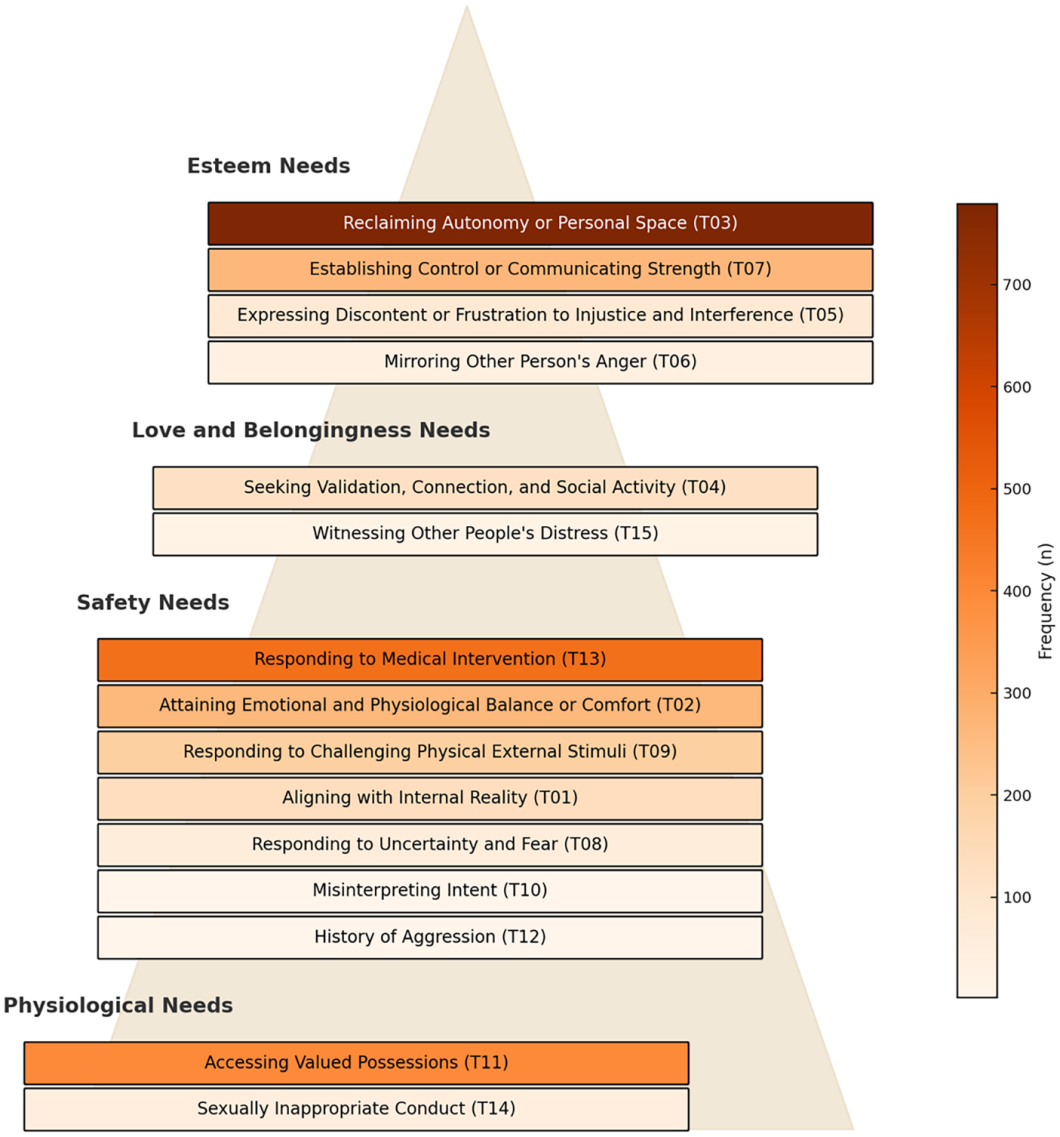
Frequency of functional features and the broad mapping of functions onto Maslow's (1943) hierarchy of needs.

The following are the 15 main themes or functional features, along with their descriptions. These themes were coded from 2555 de‐escalation logs, which include commentaries on preceding events leading up to de‐escalation (out of 2955 total logs). Each theme was considered to represent a recurring pattern of purposeful actions. There were logs with multiple coded functional features, totalling 2875. These researcher‐constructed features were descriptively presented in the order they appear in Figure 2, from bottom to top of the hierarchy. Figure 2 also illustrates the frequency with which each functional feature was observed during the intervention implementation year, with ‘Reclaiming Autonomy or Personal Space’ being the most common (*n* = 779). The mapping of functional features onto the needs in Maslow's hierarchy (1943) is also depicted in Figure 2, which situates most functions at the safety needs level.

#### 3.1.1 Physiological Needs

Behaviours within the level of physiological needs pertain to behaviours emerging from unsatisfied drives for physical comfort or stimulation, including sexually gratifying behaviours and those that support access to food and personal effects.

##### 3.1.1.1 Sexually Inappropriate Conduct (T14; *n* = 46)

This feature captures behaviours involving breaches of interpersonal boundaries, disinhibition, or fixation directed towards staff or peers, often along gender lines. Documented events included ‘taking clothes off’ (22409171148), making inappropriate or intimidating remarks against a specific gender, ‘chasing after female staff’ (32406055575), physically intrusive in gender‐designated spaces (e.g., ‘continuously attempting to go to female bedrooms’ [32504121241]), and sexually disinhibited actions.

##### 3.1.1.2 Accessing Valuable Possessions (T11; *n* = 398)

This feature encompasses behaviours driven by efforts to obtain or retain personally meaningful items such as ‘wanting access to money’ (12405231723), cigarettes, mobile phones, food, or other personal effects. Individuals frequently became distressed, verbally agitated, or escalated when access to these items was delayed, restricted, or denied. Common examples included repeated requests for smoke breaks, ‘staff called as [expletive] for not giving [the person's] phone’ (32504121241), frustration over phone charging restrictions, ‘punched fellow peer as [the person] thought they had stolen [their] clothes’ (32504081208) or personal toiletry supplies, disputes arising from television control/channel selection, and distress about food preferences or delivery delays. Sometimes, individuals became physically or verbally aggressive towards staff or peers when items were misplaced, withheld, or regulated under ward policies. A person was said to be overly focused on ‘having [their] washing, asking multiple times despite [the] washing machine still in operation’ (12408111208). This feature does not cover access requests for phone calls to connect with a specific person.

#### 3.1.2 Safety Needs

Behaviours under the level of safety include those driven by perceived or actual threats to a sense of stability, control, or personal security.

##### 3.1.2.1 History of Aggression (T12; *n* = 2)

This feature is about incidents where individuals with known patterns of prior aggression or violence exhibited behavioural escalation shortly after admission. These events typically occurred in restrictive environments, with documentation noting elevated risk related to past behaviour.

##### 3.1.2.2 Misinterpreting Intent (T10; *n* = 7)

This feature revolves around behaviours surfacing when individuals perceive harm, criticism, or control where none was intended. These events often involved agitation or verbal escalation in response to routine care interactions, such as staff introductions and staff assisting them from the bathroom to the bedroom.

##### 3.1.2.3 Responding to Uncertainty and Fear (T08; *n* = 57)

This feature frames behaviours associated with ‘anxiousness’ (22501251197) or agitation triggered by unfamiliar or unresolved situations. Individuals often became unsettled during first‐time admissions or when faced with delays in care or in receiving information. Documented examples included being ‘worried about pet’ (22412031190), being scared on the ward, and being ‘ambivalent about rehab[ilitation]’ (22412191148).

##### 3.1.2.4 Aligning With Internal Reality (T01; *n* = 133)

This feature describes behaviours reflecting experiences not shared with the immediate environment, often involving altered perceptions or beliefs. These included responses to internal stimuli such as ‘increasing hallucinations’ (12407051173; e.g., ‘patient distressed by auditory hallucinations’ [12409111217]), disorganised thinking, or enduring beliefs about safety and identity. Individuals were documented as pacing, speaking to unseen individuals, or expressing strong personal or religious convictions. For example, one person loudly declared, ‘I am God’ (32410111184) in the courtyard. Others voiced concerns about food safety or believed ‘about staff being undercover police’ (32501231223).

##### 3.1.2.5 Responding to Challenging Physical and External Stimuli (T09; *n* = 197)

This feature covers actions arising from reactions to ecological stressors within the inpatient setting. These often involve overstimulation, noise, discomfort from room temperature, and reactions to conflicts actively introduced by co‐patients, visitors, or staff. Common scenarios included agitation triggered by loud voices or music in shared spaces, with some ‘yelling at [a] peer to be quiet’ (32405098120), ‘yelling at staff in regards to music played by peers in lounge’ (32407211112), or ‘yelling, agitated as another patient woke [them] up’ (32406097265). Individuals were also observed becoming distressed after phone calls or visits from family, or when co‐patients were perceived as threatening or intrusive. This feature covers behaviours in response to antagonism, rather than deliberately being antagonistic, as in ‘co‐patients antagonised patient[,] setting [them] off’ (32405091994).

##### 3.1.2.6 Attaining Emotional and Physiological Balance or Comfort (T02; *n* = 265)

This feature captures behaviours reflecting efforts to manage overwhelming emotional or physical states. Individuals often appeared emotionally dysregulated or distressed due to internal factors such as physical pain (e.g., toothaches) and allergic reactions. Associated behavioural expressions included being ‘teary and observed low in mood for hours’ (32407151132) and self‐harming behaviours, with some expressing suicidal ideation, ‘wanting pain relief’ (12403063836), distress following poor sleep, or ‘angry being asleep all day’ (12501251236).

##### 3.1.2.7 Responding to Medical Intervention (T13; *n* = 467)

This feature depicts behaviours occurring in the context of interactions with medical professionals, medication refusal (e.g., ‘believes [they are] on too many meds’ [22409231154]), unmet expectations around medical treatment, or frustration with limited access to doctors or medical reviews (e.g., ‘demanding for the doctor to review [them] again’ [32502281213]). Preceding events frequently involved requests for specific clinicians, agitation during missed appointments, and verbalised resistance to interventions such as electroconvulsive therapy. Other examples included persistent as‐needed medication‐seeking (through IM injections) and verbal complaints regarding a mental distress diagnosis (‘began screaming and swearing at doctor during review as [they] do… not believe [they] ha[ve] a mental illness’ [32502261209]).

#### 3.1.3 Love and Belongingness Needs

Behaviours under love and belongingness needs refer to undertakings intended to uphold social connection and acceptance, which are reflected in people's actions that intervene during others' distress or seek acknowledgment and interaction from others.

##### 3.1.3.1 Witnessing Other People's Distress, Hardship, or Vulnerability (T15; *n* = 18)

This feature covers behaviours attempting to monitor, intervene in, or disrupt peer‐staff interactions or clinical procedures (e.g., ‘patient began interfering in fellow peers' care, concerning nurses’ [32404212788]), often accompanied by aggression or suspicion towards staff. Examples included ‘recording nursing staff on [their] mobile’ [32503101215] and entering peer spaces without their permission.

##### 3.1.3.2 Seeking Validation, Connection, and Social Activity (T04; *n* = 123)

This feature conveys behaviours to initiate or maintain social contact, receive acknowledgement, or alleviate isolation. Individuals frequently sought communication with family members or support persons, with documented examples including repeated requests to make phone calls (e.g., ‘wanted to ring support workers’ [32410081167]), distress over failed contact attempts (e.g., ‘yelling at nursing staff as none answered their call’ [32412311207]; ‘NIL answer when calling from family’ [32410021168]), or agitation following the end or denial of a visit (e.g., ‘yelling at staff when visitor was asked to leave ward’ [32412301203]). Other behaviours included prolonged presence at the nurses' station, verbal attempts to gain nurses' attention, or distress expressed when social needs were unmet.

#### 3.1.4 Esteem Needs

Needs that appeal to the esteem level are efforts to achieve or maintain self‐worth, independence, and perceived impartiality.

##### 3.1.4.1 Mirroring Another Person's Anger (T06; *n* = 36)

This feature refers to behaviours that appear to adopt or amplify peers' emotional tone. In some cases, patients were observed responding to the aggression of others (e.g., ‘coffee hit [them], became argumentative that [they were] assaulted by co‐patient’ [32408181159]; ‘patient was punched by [a] peer[;] in response[,] latched [o]nto peer's hair, not letting go’ [32405314261]) by yelling, joining in altercations, or becoming physically involved after an initial trigger.

##### 3.1.4.2 Expressing Discontent or Frustration to Injustice and Interference (T05; *n* = 80)

This feature centres on behaviours in which individuals respond to perceived unfairness (e.g., ‘patient escalated due to perceived injustice over wrongful admission’ [12408061147]; ‘patient increasingly preoccupied with perceived injustices by guardians’ [12409241216]), or to disruptions to their expectations. Common triggers included differential treatment received in comparison to what was seen with other patients' privileges (e.g., ‘patient saw other patients smoking and wanted access to smoking paper from locker’ [12405264534]) and dissatisfaction with communication from staff. Individuals expressed concerns about being ignored and disrespected; some indicated they would escalate the issue (e.g., by calling the police or legal representatives). Documented behaviours ranged from verbal agitation and repeated demands to expressions of distress related to institutional routines, perceived powerlessness, or broader personal grievances.

##### 3.1.4.3 Establishing Control or Communicating Strength (T07; *n* = 267)

This feature includes deliberate actions by individuals to assert social dominance and confront authority. The active use of threats, derogatory or racist remarks, and antagonism towards staff or peers characterised this feature. Individuals were frequently described as actively provoking reactions, challenging boundaries, or positioning themselves as socially powerful through verbal and physical intimidation. Behavioural manifestations included swearing, ‘thr[owing] chair and punch[ing] wall’ (32502071197), issuing threats of harm, ‘pour[ing] water over themselves’ (32502251212), ‘waking co‐patients up’ (12411281140), and using language that demeaned or degraded others, often in a manner suggestive of strategic assertion, rather than reactive escalation.

##### 3.1.4.4 Reclaiming Autonomy or Personal Space (T03; *n* = 779)

This feature includes behaviours that reflect a desire to champion personal control during hospital admission, seclusion, restraint, IM administration, and physical containment. Individuals commonly expressed distress concerning denied leave, escorted leave, police custody, community treatment order, discharge (e.g., ‘patient asked for discharge during review, punched glass on interview room door, whilst leaving room when told [they] would not be discharged’ [32502241204]), or transfer between units (e.g., ‘returned from esc[orted] leave, wanting to go to LDU [low dependency unit]’; [12403235509]). Common presentations included vocal demands for discharge, repeated pleas for leave, or attempts to abscond. Some individuals became agitated due to the restrictive environments in which they were placed, primarily in high‐observation areas.

### 3.2 Part Two: Quantitative Analyses

The narrated outcomes of the statistical and algorithmic analyses follow, including the model specification and model evaluation outcomes after hyperparameter tuning.

#### 3.2.1 Statistical Comparison of Outcomes

A small subset of categorical features demonstrated statistically significant associations with coercion‐related outcomes. Days with self‐harm (DSH) were significantly associated with days with one or more events of physical restraint (*χ* ^2^ = 4.23, *p* = 0.04), as‐needed IM psychotropics (*χ* ^2^ = 4.39, *p* = 0.04), and total restrictive practice events (*χ* ^2^ = 4.86, *p* = 0.03). Similarly, days with incidents ‘directed to property or inanimate objects’ (DPH) were significantly associated with as‐needed IM psychotropics (*χ* ^2^ = 5.29, *p* = 0.02), total restrictive practice events (*χ* ^2^ = 4.22, *p* = 0.04), and with Code Black activations (*χ* ^2^ = 4.02, *p* = 0.05). Additionally, days with noted ‘Responding to Challenging Physical External Stimuli’ (T09) were significantly associated with as‐needed IM psychotropics (*χ* ^2^ = 8.61, *p* < 0.001) and total restrictive practice events (*χ* ^2^ = 6.51, *p* = 0.01). Days coded with ‘Mirroring Another Person's Anger’ (T06) were significantly less likely to involve physical restraint (*χ* ^2^ = 5.02, *p* = 0.03). No significant group differences were observed for the continuous features (i.e., the average number of staff involved in de‐escalation [NOS] and average situational aggression before de‐escalation [PRD]) across any of the outcomes. For brevity, the full statistical outcomes are appended in the Supporting Information.

#### 3.2.2 Feature Selection Outcomes

**FIGURE 3:**
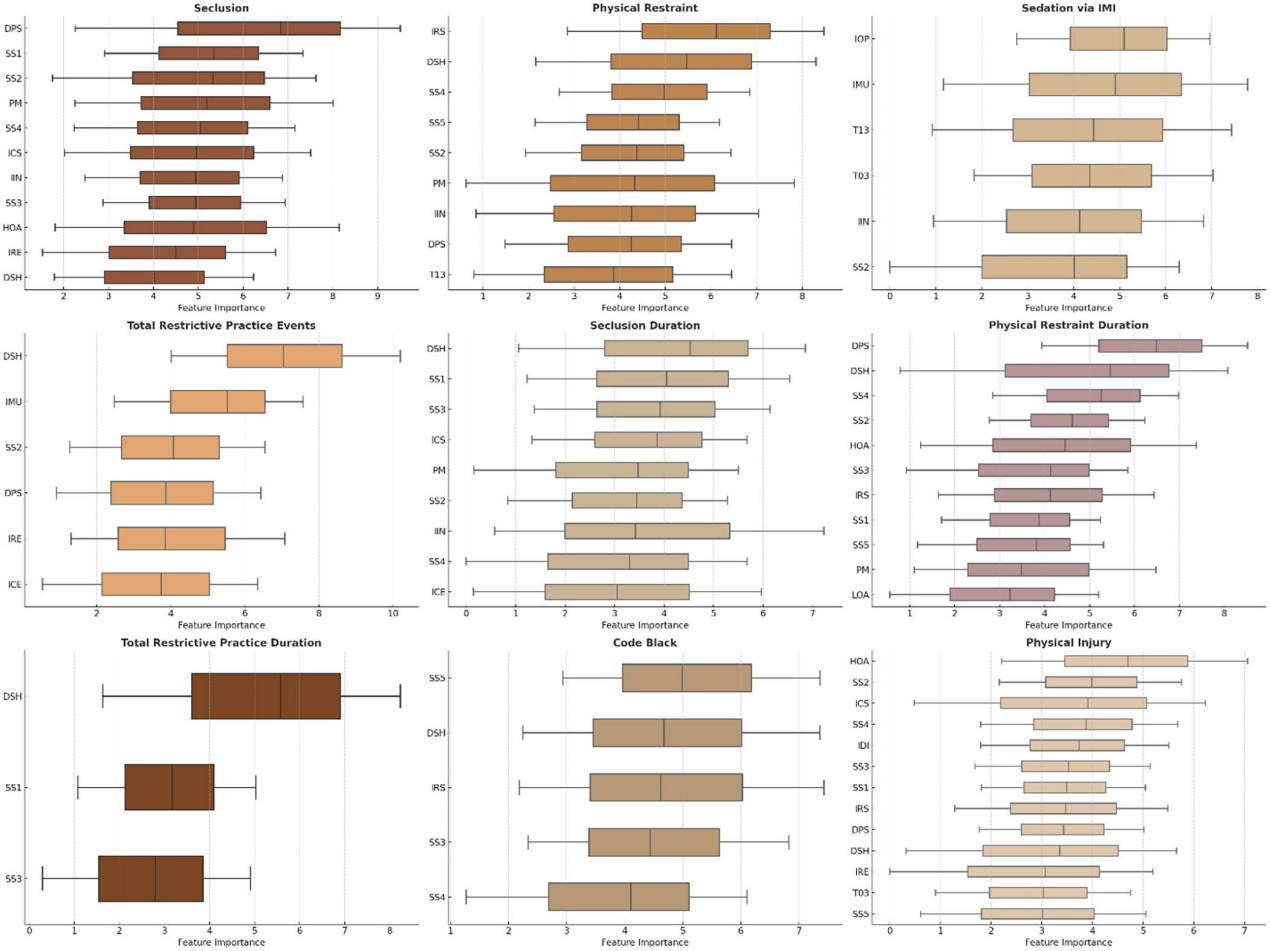
Mean, minimum, and maximum important scores of confirmed features selected through the Boruta algorithm. For brevity, importance scores of rejected and tentative features were not plotted.

Across the nine target variables, the Boruta algorithm identified between three and 13 confirmed features. The number of confirmed features per outcome was as follows: 11 for seclusion, nine for physical restraint, six for as‐needed IM psychotropics, six for total restrictive practice events, nine for seclusion duration, 11 for physical restraint duration, three for total restrictive practice duration, five for Code Black, and 13 for physical injury (see Figure 3). The confirmed features were specified in the subsequent RF regression models. In response to the overarching aim of this feature analysis, Table 2 shows the developed and evaluated predictive models.

#### 3.2.3 Hyperparameter Tuning Outcomes

**FIGURE 4:**
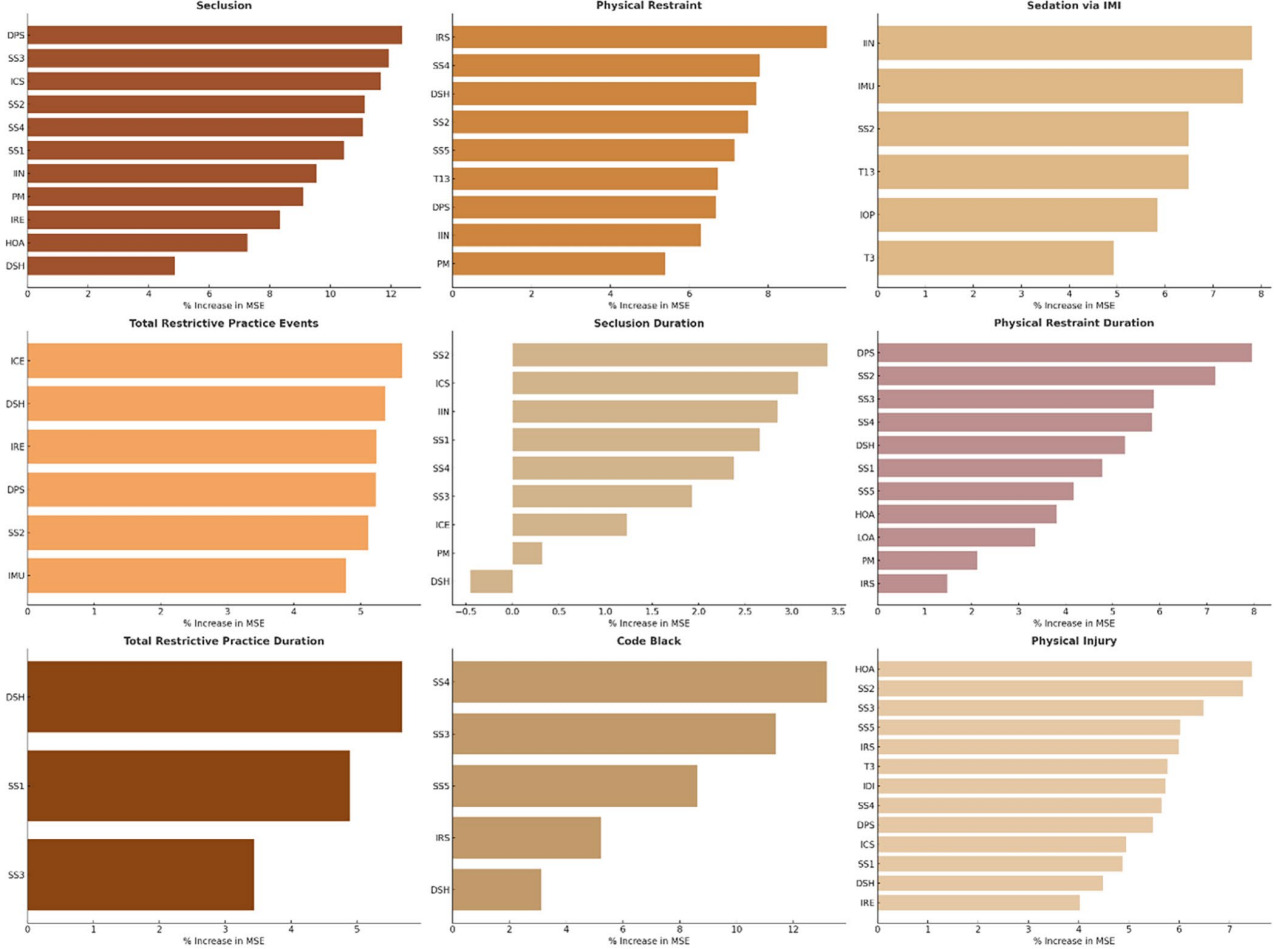
The percentage increase in mean squared error of confirmed features after hyperparameter tuning.

Distinct top‐ranking features emerged in each model for the nine target variables (see Figure 4). The most influential feature for seclusion was incidents directed at nurses (DPS). Reduced stimulus use (IRS) was the primary driver for physical restraint. The application of individualised staff time (IIN) ranked highest for as‐needed IM psychotropics, and change of environment (ICE) was the most important factor in predicting total restrictive practice events. For seclusion duration, the leading feature was the employment of ‘Identify the Issue or Problem’ (SS2). In contrast, incidents directed towards nurses (DPS) again emerged as the strongest driver of physical restraint duration. Self‐harm (DSH) was the top driver of total restrictive practice duration, and the feature associated with ‘Work Toward an Agreeable Solution’ (SS4) ranked highest for Code Black. The high observation area feature (HOA) was the most influential for physical injury.

Several features were consistently noted across multiple target variables. Incidents directed towards nurses (DPS), self‐harm (DSH), ‘Identify the Issue or Problem’ (SS2), and ‘Acknowledge and Validate’ (SS3) were among the most recurrent drivers, appearing as confirmed features in at least six outcomes. It is also worth noting that a de‐escalation event during the afternoon shift (PM) is included in the predictive models for seclusion and physical restraint events and durations. However, it was not a top driver.

#### 3.2.4 Model Evaluation Outcomes

RF regression models yielded modest predictive performance across all outcomes, with *R* ^2^ values remaining low at baseline and after hyperparameter tuning (see Table 2). Seclusion and physical restraint achieved the highest *R* ^2^ values. Hyperparameter tuning improved regression error metrics, although overall performance gains were marginal.

## 4 Discussion

This feature analysis uniquely integrates a mixed‐methods approach that draws on nurses' insights to identify key drivers of formal coercion in acute mental health units, which, to the best of the authors' knowledge, is a first in ML applications to formal coercion research set in non‐forensic adult mental health inpatient units (Daguman, Taylor, Flowers, Lakeman, and Hutchinson 2025b; Danielsen et al. 2019; Hotzy et al. 2018). This strength may both help inform and consequently prevent nurses from making decisions based on easily accessible information (Hui et al. 2021), such as the ‘history of aggression’ (T12), which did not emerged as a top driver, possibly reflecting both the underlying intervention's positive emergent impact and nurses' lesser stigmatising attitudes towards people with mental ill‐health (see Luigi et al. 2020).

The fifteen constructed functional features reflect a range of behavioural motivations that challenge the unquestioning use of invariant features as signals of formal coercion. Extending earlier works (Daffern et al. 2007; Feshbach 1964; Little et al. 2003), this functional feature set helps minimise confirmatory bias in research, where whatever aligns with the predominant explanation is reinforced (e.g., aggressive behaviour as the most important influence on formal coercion; Sailas and Wahlbeck 2005), and whatever does not is dismissed. ‘Reclaiming Autonomy or Personal Space’ offers a useful contrast: it was the most frequently noted feature but not a top driver, indicating that the comparable number of contextual, intervention, and functional features helped mitigate the tendency to deliberately find positive support for specific drivers. These features also serve as a reminder that individuals can have multiple needs simultaneously in restrictive settings, and that practitioners need to normalise people's distress to potentially defuse coercive pathways and meet the person where they are (Mulkey and Munro 2021; Price et al. 2024).

Meeting people where they are is a way of challenging the systemic focus on averting the risk that individuals will become dangerous, as many critics have long noted (Becker 1973; Foucault 1995; Rose 1985). Over‐emphasising this systemic focus has been considered a reflection of a mental health professional's limited preparation for comprehensive risk assessment (relating to stigmatising beliefs), low tolerance for work with complex issues, and fears of blame (relating to stigmatising feelings), which, in turn, contribute to formal coercion use (Haslam and Harding 2024), a form of stigmatising behaviour in itself. There is statistical support for the link between these stigma mechanisms (i.e., stigmatising beliefs, feelings, and behaviours) among health professionals (Daguman and Taylor 2025). Unfortunately, countering this stigma may be challenging in many countries, such as Australia, where generalist nursing programmes have replaced specialist mental health nursing training, leaving new nurses underprepared (Hurley et al. 2024; Warrender et al. 2024).

The functional feature ‘Responding to Challenging, Physical and External Stimuli’ was statistically associated with as‐needed IM psychotropic medications and total restrictive practice events, underlining ecological overstimulation as a key mechanism driving formal coercion (Bodryzlova et al. 2024). A systematic review revealed that ecological overstimulation, primarily through loud and repeated noise, has been associated with violent thinking, aggressive behaviour, absconding, sleep interruption, and reduced stress tolerance (Weber et al. 2022), which, in turn, can be observed by nurses as signs of escalating risk. Conversely, unit designs that offer privacy and calm spaces are associated with lower coercion rates (Oostermeijer et al. 2021). Scaling up inpatient infrastructures is then warranted and justified, especially in parts of NSW, Australia, where outdated custodial physical layouts contain several people under heightened monitoring in a single room. This layout is an example of a panopticon (Salzmann‐Erikson and Eriksson 2012), a system of control in which people self‐regulate in response to the constant threat of surveillance, offering no refuge from distress.

Since both ‘Responding to Challenging, Physical and External Stimuli’ and the usual function of formal coercion (i.e., ensuring long‐term recovery and safety; Chieze et al. 2021) fall under the level of safety needs (Maslow 1943), there is some support to say that specific values hold more weight than others for nurses. Behaviours with a function of ‘Responding to Challenging, Physical and External Stimuli’ may reflect a person's attempt to regain personal safety, but can be a mismatch to the inpatient unit's goal for collective safety, which may lead to formal coercion. This finding does not contradict Maslow's model, as needs can emerge fluidly and coexist even in a ‘reversed order’ (Feist et al. 2021, 284). A mismatch between a person's behaviour and the inpatient unit's goal further suggests that meeting a person's needs depends on the broader care environment, where value arrangements determine which needs and perspectives are given primacy. Fairness in the provision of care (procedural justice) depends on whether people's experiences are being heard and taken seriously (testimonial justice; Lakeman 2010). Understanding the values of mental health services may then help understand how mental health practices either reinforce or reduce the use of formal coercion.

Despite the widened pool of functional features, incidents directed towards nurses and self‐harm remained among the top formal coercion drivers, with self‐harm also demonstrating significant statistical associations. Externally validating these findings is complex, as the literature offers mixed outcomes. Although the finding on incidents directed towards nurses aligns with a recent NSW feature analysis (Daguman, Taylor, Flowers, Lakeman, and Hutchinson 2025b), the self‐harm finding differs from those reported in Paris psychiatric centres on seclusion and restraint durations (Dauriac‐Le Masson et al. 2024). One explanation may be the dynamics in such incidents, where a person's reactivity, past trauma, and situational stressors intersect (Delaney 2025). In the same vein, given that de‐escalation is a collective nursing practice (Daguman et al. 2026), these two features may have influenced the clinical reasoning of nursing teams (Beeber et al. 2025; Conlon et al. 2024) that judged less coercive alternatives as not readily accessible or impractical. Supporting nurses' reflexivity around formal coercion decisions may then be helpful in ‘decompressing’ (Maguire et al. 2025, 1) or restoring damaged collegial and person‐nurse relationships (Hutchinson 2009). There is evidence to say that the conduct of strengths‐based restrictive practice reviews reduced the seclusion rate in an acute mental health unit (Daguman, Taylor, Flowers, Lakeman, and Hutchinson 2025c).

The top algorithmic driver on many target variables was interventional. In particular, the reduced stimulus emerged as the strongest driver of physical restraint and changes in the environment for total restrictive practice events. These findings align with the support‐control continuum, where ‘routine instruction to low stimulus areas’ (Price et al. 2018, 201) can be ‘escalatory.’ In contrast, soothing new environments and stimuli can help regulate sensory arousal (Kandlur et al. 2023). On the other hand, individualised staff time was the top feature for as‐needed IM psychotropics, which relates to professional guidelines recommending exhausting such engagement alongside oral as‐needed medication to avoid the more coercive, as‐needed IM in response to acute behavioural disturbance (Curry et al. 2023; NSW Government Agency for Clinical Innovation 2015). In contrast, a study in Australia found no significant association between one‐on‐one nursing and the increased odds of restrictive practices (Maguire et al. 2018). These findings emphasise that reduced stimulus, a change in environment, and individualised staff time can either reduce or increase coercion risks, depending on their practical and relational context.

Among the interventional features, ‘Identify the Issue or Problem’ was the most influential for seclusion duration, whereas ‘Work Towards an Agreeable Solution’ was the strongest driver for Code Black. Both features also appeared as drivers across other target variables, suggesting that some relational practices may act more as turning points within ‘a cascade of effects’ (Bergström et al. 2012, 2) once events have already begun to deteriorate and may mean that ‘the point that the model of strict adherence to best practice guidelines needs to be complemented’ (p. 3). Fear further complicates these interactions (Goodman et al. 2020), as nurses' emotional response can shape negative views about de‐escalation and prompt premature use of formal coercion. For ‘Work Towards an Agreeable Solution,’ Andersson et al. (2020) argued that nurses may use negotiation to preserve a person's autonomy. However, these negotiations may also reflect power imbalances and informal coercion that could become formal. Well‐meaning actions, especially in heightened contexts, may then risk becoming steps towards formal coercion when misperceived or misapplied.

Despite hyperparameter tuning, the RF regressions consistently yielded low *R* ^2^ values across target variables, reflecting the complexity of the constructs under study. These findings align with broader ML findings in psychiatry, where many individual predictors have limited explanatory power (Pearson et al. 2019), and the use of restrictive practices is a context‐sensitive outcome (Belayneh et al. 2025). Moreover, staff dynamics, leadership changes in mental health services, and other culture‐related factors can significantly influence formal coercion, particularly in the context of seclusion (Boumans et al. 2015). These contextual drivers are rarely captured in routine structured data, and if added as items to purpose‐built measures, can also be cost‐inhibitive for nurses to complete, given the busy nature of work in mental health inpatient units (Bowers et al. 2015). However, it has been argued that focusing on individual‐level features can create a reasoning bias among intervention evaluators, leading them to attribute failures in intervention research to people receiving care, rather than to mental health services (Daguman et al. 2024). Overall, innumerable, unexamined drivers could contribute to predicting and explaining formal coercion.

This feature analysis has limitations. First, while functional features were systematically coded from nurse‐recorded logs, further factor analysis could clarify how well these items reflect underlying constructs. Second, the analysis was limited to three inpatient units without external validation. However, some context‐sensitivity is expected in ML applications (Wu and Fukui 2024). Third, involving frontline staff and people with lived experience of coercion in interpreting findings may enhance the model's explanatory depth and practical relevance. Lastly, of course, there are limitations to Maslow's theory of motivation, including the lack of supporting evidence that disconfirms its propositions (falsifiability; Feist et al. 2021). Nonetheless, it allows one to see the person as a whole.

## 5 Conclusions

This feature analysis demonstrates that the use of formal coercion in acute mental health units cannot be reduced to a single cause or to invariant risk signals. Rather, decisions around their use surface from a dynamic mix of environmental, motivational, and practical factors. A range of features served as ‘turning points,’ indicating that coercion is not inevitable. Nonetheless, a vast, viable space still exists for alternative, less coercive responses. The predictive power of the algorithmic models was modest; however, the insights gained from this feature analysis provide support for the importance of undertaking more thoughtful, context‐sensitive interpretations of machine learning outputs in mental health research.

## 6 Relevance for Clinical Practice

It is justifiable to make a quantitative statement that nurses' use of formal coercion is not solely a response to invariant individual‐level features. More importantly, some of the causes of formal coercion use are outside the broader sphere of nursing competencies, which include not only being aggression management therapists, but also being advocates of people receiving care, physical health therapists, psychotherapists, psychopharmacological therapists, and relationship‐focused therapists (Hurley and Lakeman 2021). Another practical point that emerged is the need to develop and apply (or mimic) physical infrastructure designs that are not conducive to ecological overstimulation, which can arguably be best addressed by senior management and leaders responsible for dispensing financial resources to acute inpatient services. There is also a need to invest in staff support and restorative practices, so that nurses can reflect on their formal coercion application decisions. Lastly, preventing formal coercion still depends on enhancing nurses' relational capabilities. So, it is critical that their formal education and professional training prepare them to transform ‘turning points’ into opportunities for noncoercive responses and support.

## Author Contributions

**Esario IV Daguman:** conceptualisation, methodology, software, formal analysis, investigation, resources, validation, data curation, writing – original draft, and visualisation. **Jacqui Yoxall:** resources, and writing – review and editing. **Richard Lakeman:** resources, and writing – review and editing. **Marie Hutchinson:** conceptualisation, methodology, resources, writing – review and editing, funding acquisition, and supervision.

## Funding

This feature analysis is part of a larger research project funded by Southern Cross University and the Translational Research Grant Scheme from the NSW Office for Health and Medical Research.

## Conflicts of Interest

Esario IV Daguman is supported by a PhD scholarship jointly funded by Southern Cross University and the Translational Research Grant Scheme of the NSW Office for Health and Medical Research. Neither funding bodies contributed to the conceptualisation, conduct, analyses, reporting, interpretation, and writing of this feature analysis. The views expressed in this research are those of the authors and do not necessarily represent those of the funding bodies, the organisational affiliations of the authors, nor those of the research and implementation partners of the larger project in which this research is situated.

## Supporting information


**Data S1:** The brief descriptions of the interventional features and the outcomes of the bivariate inferential statistics for this feature analysis.

## Data Availability

The datasets gathered, analysed, and interpreted for this feature analysis are not publicly available, due to conditions in the ethical approvals obtained.
