# Supplementary material for: Functional, Contextual, and Interventional Drivers of Formal Coercion in Acute Mental Health Units: A Feature Analysis
Source: Int J Ment Health Nurs. 2026 Feb 24;35(1):e70240. doi: 10.1111/inm.70240 (PMC12930232; doi:10.1111/inm.70240)
Supplement: Supplementary file 1 — Data S1: The brief descriptions of the interventional features and the outcomes of the bivariate inferential statistics for this feature analysis. [file INM-35-0-s001.docx]

# Supplementary Materials

## Table of Contents

[Supplementary Materials 1](#_Toc212460669)

[Table of Contents 1](#_Toc212460670)

[Description of Interventional Features 2](#_Toc212460671)

[Outcomes of Bivariate Inferential Statistics for Categorical Predictors 6](#_Toc212460672)

[Outcomes of Bivariate Inferential Statistics for Continuous Predictors 15](#_Toc212460673)

## Description of interventional features

***SS1 – Staff emotional self-regulation or self-management***
Nurses engage in actions to adjust their own emotional state during interactions with individuals they support. This intervention may include altering tone, breathing, posture, to express a neutral or calm presence.

***SS2 – Identify the issue or problem***
Nurses assist the person in articulating, recognising, or renaming the source of their distress or conflict. This intervention may cover asking questions, active listening, building rapport, and clarifying what triggered the current situation.

***SS3 – Acknowledge and validate***
Nurses respond by naming or reflecting the person’s feelings, thoughts, or concerns. This intervention can include verbal expressions of understanding or simple recognition of the person's emotional state, refocusing of a discussion, and labelling emotions expressed by the person.

***SS4 – Work toward agreeable solution***
Nurses and the person take part in dialogue to explore the possible next actions that could support the resolution of the identified concern. This intervention may involve proposing a range of viable options, a discussion of the person’s preferences, or the attainment of a point of mutual agreement.

**SS5 – Establish expected behaviour**
Nurses clearly communicate reasonable expectations or boundaries regarding behaviour within the inpatient unit. This intervention may include kind reminders about rules, roles, or safety policies and procedures relevant to the incumbent situation.

**IDI – Distraction**
Nurses introduce alternative topics or activities to enable the person to think of something else other than the source of distress or conflict. This intervention may involve casual conversation, humour, or offering materials to occupy the hands.

**IRE – Redirection**
Nurses guide the person to a different purpose. This often occurs through brief verbal prompts or non-verbal cues intended to interrupt escalating behaviours.

**ICE – Change environment**
Nurses accompany or direct the person to a different physical location within the inpatient unit. The new setting may have different sensory characteristics or involve fewer people.

**IRS – Reduced stimulus**
Nurses lower or remove environmental inputs such as noise, light, or tactile stimuli. This intervention may include turning off devices, dimming lights, or limiting the number of people present.

**IMU – Music**
Nurses provide access to musical pieces through the use of headphones, speakers, or audiovisual devices. The music may be selected by the person, pre-set by the inpatient unit, or may be used in isolation or in conjunction with other sensory tools.

**IOP – As-needed oral medication**

Nurses administer medication that has been prescribed to be taken as needed within the incumbent situation. This intervention follows a clinical decision and is governed by, and documented according to, policy.

**IIN – Individualised staff time**
Nurses spend time with the person outside of group or routine care activities. This intervention may involve sitting together, engaging in light conversation, or assisting with personal hygiene tasks.

**IFD – Food and drinks**
Nurses offer or provide snacks, light meals, refreshments or beverages to the person. This intervention can occur during routine times or be initiated in response to distress or agitation.

**ICS – Culturally sensitive care**
Nurses make available, or support access to, cultural, spiritual, or identity-based practices or items. This intervention may include prayer space, traditional music, or culturally familiar foods.

**ISM – Sensory modulation**
Nurses provide materials or equipment intended to engage the person’s senses. Items may include weighted objects (e.g., weighted blankets), textured items, essential oils, or massage chairs.

**IPC – Phone call**
Nurses support the person to access telephonic devices to make or receive calls. The call may involve conversation with family, carers, supporters, legal representatives, or other external contacts (e.g., banks), and may be supervised or unsupervised.

## Outcomes of bivariate inferential statistics for categorical predictors

Refer to Table 1 for feature and target variable codes, feature category, and code description.

| **Feature** | **Target Variable** | **Test** | **Feature and Target Variable Present** | **Absent Feature, Present Target Variable** | **Present Feature, Target Variable Absent** | **Feature and Target Variable Absent** | **Statistic** | ***p*** |
| --- | --- | --- | --- | --- | --- | --- | --- | --- |
| AM | SEC | Chi-square | 46 | 5 | 269 | 45 | 0.43 | 0.51 |
| AM | RES | Chi-square | 77 | 13 | 238 | 37 | 0.00 | 0.95 |
| AM | SED | Chi-square | 240 | 33 | 75 | 17 | 1.87 | 0.17 |
| AM | RPC | Chi-square | 245 | 33 | 70 | 17 | 2.68 | 0.10 |
| AM | CDB | Chi-square | 78 | 10 | 237 | 40 | 0.31 | 0.58 |
| AM | TPI | Fisher | 7 | 1 | 308 | 49 |  | 1.00 |
| PM | SEC | Chi-square | 39 | 12 | 220 | 94 | 0.59 | 0.44 |
| PM | RES | Chi-square | 68 | 22 | 191 | 84 | 0.95 | 0.33 |
| PM | SED | Chi-square | 197 | 76 | 62 | 30 | 0.55 | 0.46 |
| PM | RPC | Chi-square | 201 | 77 | 58 | 29 | 0.77 | 0.38 |
| PM | CDB | Chi-square | 62 | 26 | 197 | 80 | 0.00 | 1.00 |
| PM | TPI | Fisher | 6 | 2 | 253 | 104 |  | 1.00 |
| Night | SEC | Chi-square | 13 | 38 | 94 | 220 | 0.23 | 0.63 |
| Night | RES | Chi-square | 22 | 68 | 85 | 190 | 1.07 | 0.30 |
| Night | SED | Chi-square | 84 | 189 | 23 | 69 | 0.84 | 0.36 |
| Night | RPC | Chi-square | 84 | 194 | 23 | 64 | 0.29 | 0.59 |
| Night | CDB | Chi-square | 27 | 61 | 80 | 197 | 0.04 | 0.85 |
| Night | TPI | Fisher | 4 | 4 | 103 | 254 |  | 0.24 |
| HOA | SEC | Chi-square | 38 | 13 | 216 | 98 | 0.43 | 0.51 |
| HOA | RES | Chi-square | 63 | 27 | 191 | 84 | 0.00 | 1.00 |
| HOA | SED | Chi-square | 193 | 80 | 61 | 31 | 0.44 | 0.51 |
| HOA | RPC | Chi-square | 197 | 81 | 57 | 30 | 0.66 | 0.42 |
| HOA | CDB | Chi-square | 65 | 23 | 189 | 88 | 0.75 | 0.39 |
| HOA | TPI | Fisher | 7 | 1 | 247 | 110 |  | 0.44 |
| LOA | SEC | Chi-square | 42 | 9 | 270 | 44 | 0.22 | 0.64 |
| LOA | RES | Chi-square | 75 | 15 | 237 | 38 | 0.24 | 0.62 |
| LOA | SED | Chi-square | 236 | 37 | 76 | 16 | 0.54 | 0.46 |
| LOA | RPC | Chi-square | 239 | 39 | 73 | 14 | 0.09 | 0.76 |
| LOA | CDB | Chi-square | 72 | 16 | 240 | 37 | 0.89 | 0.34 |
| LOA | TPI | Fisher | 8 | 0 | 304 | 53 |  | 0.61 |
| DCA | SEC | Chi-square | 17 | 34 | 99 | 215 | 0.01 | 0.93 |
| DCA | RES | Chi-square | 25 | 65 | 91 | 184 | 0.65 | 0.42 |
| DCA | SED | Chi-square | 87 | 186 | 29 | 63 | 0.00 | 1.00 |
| DCA | RPC | Chi-square | 89 | 189 | 27 | 60 | 0.00 | 0.97 |
| DCA | CDB | Chi-square | 32 | 56 | 84 | 193 | 0.86 | 0.35 |
| DCA | TPI | Fisher | 0 | 8 | 116 | 241 |  | 0.06 |
| DAP | SEC | Chi-square | 25 | 26 | 153 | 161 | 0.00 | 1.00 |
| DAP | RES | Chi-square | 42 | 48 | 136 | 139 | 0.11 | 0.74 |
| DAP | SED | Chi-square | 136 | 137 | 42 | 50 | 0.33 | 0.57 |
| DAP | RPC | Chi-square | 137 | 141 | 41 | 46 | 0.05 | 0.82 |
| DAP | CDB | Chi-square | 49 | 39 | 129 | 148 | 1.87 | 0.17 |
| DAP | TPI | Fisher | 3 | 5 | 175 | 182 |  | 0.72 |
| DSH | SEC | Chi-square | 27 | 24 | 172 | 142 | 0.01 | 0.93 |
| DSH | RES | Chi-square | 58 | 32 | 141 | 134 | 4.23 | **0.04** |
| DSH | SED | Chi-square | 158 | 115 | 41 | 51 | 4.39 | **0.04** |
| DSH | RPC | Chi-square | 161 | 117 | 38 | 49 | 4.86 | **0.03** |
| DSH | CDB | Chi-square | 49 | 39 | 150 | 127 | 0.02 | 0.90 |
| DSH | TPI | Fisher | 5 | 3 | 194 | 163 |  | 0.73 |
| DPS | SEC | Fisher | 45 | 6 | 296 | 18 |  | 0.12 |
| DPS | RES | Chi-square | 81 | 9 | 260 | 15 | 1.60 | 0.21 |
| DPS | SED | Chi-square | 253 | 20 | 88 | 4 | 0.57 | 0.45 |
| DPS | RPC | Chi-square | 258 | 20 | 83 | 4 | 0.37 | 0.55 |
| DPS | CDB | Chi-square | 81 | 7 | 260 | 17 | 0.12 | 0.73 |
| DPS | TPI | Fisher | 8 | 0 | 333 | 24 |  | 1.00 |
| DPV | SEC | Chi-square | 5 | 46 | 32 | 282 | 0.00 | 1.00 |
| DPV | RES | Chi-square | 11 | 79 | 26 | 249 | 0.31 | 0.58 |
| DPV | SED | Chi-square | 31 | 242 | 6 | 86 | 1.27 | 0.26 |
| DPV | RPC | Chi-square | 31 | 247 | 6 | 81 | 0.89 | 0.35 |
| DPV | CDB | Chi-square | 10 | 78 | 27 | 250 | 0.06 | 0.81 |
| DPV | TPI | Fisher | 0 | 8 | 37 | 320 |  | 1.00 |
| DVP | SEC | Fisher | 0 | 51 | 3 | 311 |  | 1.00 |
| DVP | RES | Fisher | 1 | 89 | 2 | 273 |  | 0.57 |
| DVP | SED | Fisher | 3 | 270 | 0 | 92 |  | 0.58 |
| DVP | RPC | Fisher | 3 | 275 | 0 | 87 |  | 1.00 |
| DVP | CDB | Fisher | 0 | 88 | 3 | 274 |  | 1.00 |
| DVP | TPI | Fisher | 0 | 8 | 3 | 354 |  | 1.00 |
| DVS | SEC | Fisher | 1 | 50 | 11 | 303 |  | 1.00 |
| DVS | RES | Fisher | 3 | 87 | 9 | 266 |  | 1.00 |
| DVS | SED | Fisher | 11 | 262 | 1 | 91 |  | 0.31 |
| DVS | RPC | Fisher | 11 | 267 | 1 | 86 |  | 0.31 |
| DVS | CDB | Fisher | 5 | 83 | 7 | 270 |  | 0.17 |
| DVS | TPI | Fisher | 0 | 8 | 12 | 345 |  | 1.00 |
| DPH | SEC | Chi-square | 13 | 38 | 54 | 260 | 1.50 | 0.22 |
| DPH | RES | Chi-square | 20 | 70 | 47 | 228 | 0.87 | 0.35 |
| DPH | SED | Chi-square | 58 | 215 | 9 | 83 | 5.29 | **0.02** |
| DPH | RPC | Chi-square | 58 | 220 | 9 | 78 | 4.22 | **0.04** |
| DPH | CDB | Chi-square | 23 | 65 | 44 | 233 | 4.02 | **0.05** |
| DPH | TPI | Fisher | 0 | 8 | 67 | 290 |  | 0.36 |
| DEU | SEC | Fisher | 51 | 0 | 313 | 1 |  | 1.00 |
| DEU | RES | Fisher | 90 | 0 | 274 | 1 |  | 1.00 |
| DEU | SED | Fisher | 273 | 0 | 91 | 1 |  | 0.25 |
| DEU | RPC | Fisher | 278 | 0 | 86 | 1 |  | 0.24 |
| DEU | CDB | Fisher | 88 | 0 | 276 | 1 |  | 1.00 |
| DEU | TPI | Fisher | 8 | 0 | 356 | 1 |  | 1.00 |
| SS1 | SEC | Fisher | 50 | 1 | 306 | 8 |  | 1.00 |
| SS1 | RES | Fisher | 89 | 1 | 267 | 8 |  | 0.46 |
| SS1 | SED | Fisher | 266 | 7 | 90 | 2 |  | 1.00 |
| SS1 | RPC | Fisher | 271 | 7 | 85 | 2 |  | 1.00 |
| SS1 | CDB | Fisher | 85 | 3 | 271 | 6 |  | 0.46 |
| SS1 | TPI | Fisher | 8 | 0 | 348 | 9 |  | 1.00 |
| SS2 | SEC | Fisher | 50 | 1 | 308 | 6 |  | 1.00 |
| SS2 | RES | Fisher | 88 | 2 | 270 | 5 |  | 0.68 |
| SS2 | SED | Fisher | 268 | 5 | 90 | 2 |  | 1.00 |
| SS2 | RPC | Fisher | 273 | 5 | 85 | 2 |  | 0.67 |
| SS2 | CDB | Fisher | 87 | 1 | 271 | 6 |  | 1.00 |
| SS2 | TPI | Fisher | 8 | 0 | 350 | 7 |  | 1.00 |
| SS3 | SEC | Fisher | 51 | 0 | 309 | 5 |  | 1.00 |
| SS3 | RES | Fisher | 89 | 1 | 271 | 4 |  | 1.00 |
| SS3 | SED | Fisher | 271 | 2 | 89 | 3 |  | 0.10 |
| SS3 | RPC | Fisher | 276 | 2 | 84 | 3 |  | 0.09 |
| SS3 | CDB | Fisher | 87 | 1 | 273 | 4 |  | 1.00 |
| SS3 | TPI | Fisher | 8 | 0 | 352 | 5 |  | 1.00 |
| SS4 | SEC | Fisher | 49 | 2 | 307 | 7 |  | 0.37 |
| SS4 | RES | Fisher | 88 | 2 | 268 | 7 |  | 1.00 |
| SS4 | SED | Fisher | 267 | 6 | 89 | 3 |  | 0.70 |
| SS4 | RPC | Fisher | 272 | 6 | 84 | 3 |  | 0.45 |
| SS4 | CDB | Fisher | 86 | 2 | 270 | 7 |  | 1.00 |
| SS4 | TPI | Fisher | 8 | 0 | 348 | 9 |  | 1.00 |
| SS5 | SEC | Fisher | 50 | 1 | 295 | 19 |  | 0.33 |
| SS5 | RES | Fisher | 86 | 4 | 259 | 16 |  | 0.79 |
| SS5 | SED | Chi-square | 260 | 13 | 85 | 7 | 0.60 | 0.44 |
| SS5 | RPC | Fisher | 265 | 13 | 80 | 7 |  | 0.28 |
| SS5 | CDB | Fisher | 86 | 2 | 259 | 18 |  | 0.18 |
| SS5 | TPI | Fisher | 8 | 0 | 337 | 20 |  | 1.00 |
| IDI | SEC | Chi-square | 36 | 15 | 244 | 70 | 0.88 | 0.35 |
| IDI | RES | Chi-square | 70 | 20 | 210 | 65 | 0.02 | 0.90 |
| IDI | SED | Chi-square | 212 | 61 | 68 | 24 | 0.35 | 0.55 |
| IDI | RPC | Chi-square | 215 | 63 | 65 | 22 | 0.13 | 0.72 |
| IDI | CDB | Chi-square | 69 | 19 | 211 | 66 | 0.08 | 0.77 |
| IDI | TPI | Fisher | 6 | 2 | 274 | 83 |  | 1.00 |
| IRE | SEC | Fisher | 50 | 1 | 301 | 13 |  | 0.70 |
| IRE | RES | Fisher | 89 | 1 | 262 | 13 |  | 0.20 |
| IRE | SED | Fisher | 264 | 9 | 87 | 5 |  | 0.36 |
| IRE | RPC | Fisher | 269 | 9 | 82 | 5 |  | 0.34 |
| IRE | CDB | Fisher | 84 | 4 | 267 | 10 |  | 0.75 |
| IRE | TPI | Fisher | 8 | 0 | 343 | 14 |  | 1.00 |
| ICE | SEC | Chi-square | 33 | 18 | 210 | 104 | 0.02 | 0.89 |
| ICE | RES | Chi-square | 61 | 29 | 182 | 93 | 0.02 | 0.88 |
| ICE | SED | Chi-square | 181 | 92 | 62 | 30 | 0.00 | 0.95 |
| ICE | RPC | Chi-square | 184 | 94 | 59 | 28 | 0.02 | 0.88 |
| ICE | CDB | Chi-square | 61 | 27 | 182 | 95 | 0.25 | 0.62 |
| ICE | TPI | Fisher | 5 | 3 | 238 | 119 |  | 1.00 |
| IRS | SEC | Chi-square | 33 | 18 | 203 | 111 | 0.00 | 1.00 |
| IRS | RES | Chi-square | 60 | 30 | 176 | 99 | 0.11 | 0.74 |
| IRS | SED | Chi-square | 178 | 95 | 58 | 34 | 0.06 | 0.80 |
| IRS | RPC | Chi-square | 180 | 98 | 56 | 31 | 0.00 | 1.00 |
| IRS | CDB | Chi-square | 57 | 31 | 179 | 98 | 0.00 | 1.00 |
| IRS | TPI | Fisher | 6 | 2 | 230 | 127 |  | 0.72 |
| IMU | SEC | Chi-square | 19 | 32 | 107 | 207 | 0.08 | 0.78 |
| IMU | RES | Chi-square | 35 | 55 | 91 | 184 | 0.77 | 0.38 |
| IMU | SED | Chi-square | 98 | 175 | 28 | 64 | 0.68 | 0.41 |
| IMU | RPC | Chi-square | 100 | 178 | 26 | 61 | 0.83 | 0.36 |
| IMU | CDB | Chi-square | 32 | 56 | 94 | 183 | 0.08 | 0.77 |
| IMU | TPI | Fisher | 1 | 7 | 125 | 232 |  | 0.27 |
| IOP | SEC | Chi-square | 42 | 9 | 246 | 68 | 0.22 | 0.64 |
| IOP | RES | Chi-square | 75 | 15 | 213 | 62 | 1.08 | 0.30 |
| IOP | SED | Chi-square | 218 | 55 | 70 | 22 | 0.38 | 0.54 |
| IOP | RPC | Chi-square | 223 | 55 | 65 | 22 | 0.90 | 0.34 |
| IOP | CDB | Chi-square | 70 | 18 | 218 | 59 | 0.00 | 0.99 |
| IOP | TPI | Fisher | 5 | 3 | 283 | 74 |  | 0.37 |
| IIN | SEC | Chi-square | 33 | 18 | 194 | 120 | 0.06 | 0.81 |
| IIN | RES | Chi-square | 54 | 36 | 173 | 102 | 0.14 | 0.71 |
| IIN | SED | Chi-square | 171 | 102 | 56 | 36 | 0.03 | 0.86 |
| IIN | RPC | Chi-square | 174 | 104 | 53 | 34 | 0.02 | 0.88 |
| IIN | CDB | Chi-square | 54 | 34 | 173 | 104 | 0.00 | 0.95 |
| IIN | TPI | Fisher | 7 | 1 | 220 | 137 |  | 0.27 |
| IFD | SEC | Chi-square | 11 | 40 | 67 | 247 | 0.00 | 1.00 |
| IFD | RES | Chi-square | 20 | 70 | 58 | 217 | 0.01 | 0.94 |
| IFD | SED | Chi-square | 61 | 212 | 17 | 75 | 0.40 | 0.53 |
| IFD | RPC | Chi-square | 62 | 216 | 16 | 71 | 0.39 | 0.53 |
| IFD | CDB | Chi-square | 17 | 71 | 61 | 216 | 0.15 | 0.70 |
| IFD | TPI | Fisher | 3 | 5 | 75 | 282 |  | 0.38 |
| ICS | SEC | Chi-square | 42 | 9 | 283 | 31 | 1.98 | 0.16 |
| ICS | RES | Chi-square | 84 | 6 | 241 | 34 | 1.71 | 0.19 |
| ICS | SED | Chi-square | 246 | 27 | 79 | 13 | 0.87 | 0.35 |
| ICS | RPC | Chi-square | 250 | 28 | 75 | 12 | 0.60 | 0.44 |
| ICS | CDB | Chi-square | 80 | 8 | 245 | 32 | 0.20 | 0.65 |
| ICS | TPI | Fisher | 8 | 0 | 317 | 40 |  | 0.61 |
| ISM | SEC | Chi-square | 16 | 35 | 76 | 238 | 0.85 | 0.36 |
| ISM | RES | Chi-square | 24 | 66 | 68 | 207 | 0.05 | 0.82 |
| ISM | SED | Chi-square | 73 | 200 | 19 | 73 | 1.05 | 0.31 |
| ISM | RPC | Chi-square | 74 | 204 | 18 | 69 | 0.94 | 0.33 |
| ISM | CDB | Chi-square | 25 | 63 | 67 | 210 | 0.43 | 0.51 |
| ISM | TPI | Fisher | 1 | 7 | 91 | 266 |  | 0.69 |
| IPC | SEC | Fisher | 2 | 49 | 20 | 294 |  | 0.75 |
| IPC | RES | Chi-square | 6 | 84 | 16 | 259 | 0.00 | 0.97 |
| IPC | SED | Chi-square | 17 | 256 | 5 | 87 | 0.00 | 0.98 |
| IPC | RPC | Chi-square | 17 | 261 | 5 | 82 | 0.00 | 1.00 |
| IPC | CDB | Chi-square | 6 | 82 | 16 | 261 | 0.01 | 0.92 |
| IPC | TPI | Fisher | 0 | 8 | 22 | 335 |  | 1.00 |
| IMS | SEC | Chi-square | 13 | 38 | 63 | 251 | 0.49 | 0.48 |
| IMS | RES | Chi-square | 18 | 72 | 58 | 217 | 0.01 | 0.94 |
| IMS | SED | Chi-square | 57 | 216 | 19 | 73 | 0.00 | 1.00 |
| IMS | RPC | Chi-square | 59 | 219 | 17 | 70 | 0.03 | 0.85 |
| IMS | CDB | Chi-square | 19 | 69 | 57 | 220 | 0.00 | 0.96 |
| IMS | TPI | Fisher | 2 | 6 | 74 | 283 |  | 0.67 |
| T1 | SEC | Chi-square | 15 | 36 | 85 | 229 | 0.03 | 0.86 |
| T1 | RES | Chi-square | 20 | 70 | 80 | 195 | 1.28 | 0.26 |
| T1 | SED | Chi-square | 73 | 200 | 27 | 65 | 0.12 | 0.73 |
| T1 | RPC | Chi-square | 73 | 205 | 27 | 60 | 0.54 | 0.46 |
| T1 | CDB | Chi-square | 26 | 62 | 74 | 203 | 0.15 | 0.70 |
| T1 | TPI | Fisher | 0 | 8 | 100 | 257 |  | 0.11 |
| T2 | SEC | Chi-square | 21 | 30 | 133 | 181 | 0.00 | 1.00 |
| T2 | RES | Chi-square | 38 | 52 | 116 | 159 | 0.00 | 1.00 |
| T2 | SED | Chi-square | 120 | 153 | 34 | 58 | 1.11 | 0.29 |
| T2 | RPC | Chi-square | 123 | 155 | 31 | 56 | 1.68 | 0.20 |
| T2 | CDB | Chi-square | 38 | 50 | 116 | 161 | 0.01 | 0.93 |
| T2 | TPI | Fisher | 4 | 4 | 150 | 207 |  | 0.73 |
| T3 | SEC | Chi-square | 37 | 14 | 213 | 101 | 0.26 | 0.61 |
| T3 | RES | Chi-square | 67 | 23 | 183 | 92 | 1.61 | 0.20 |
| T3 | SED | Chi-square | 190 | 83 | 60 | 32 | 0.43 | 0.51 |
| T3 | RPC | Chi-square | 194 | 84 | 56 | 31 | 0.67 | 0.41 |
| T3 | CDB | Chi-square | 66 | 22 | 184 | 93 | 1.90 | 0.17 |
| T3 | TPI | Fisher | 5 | 3 | 245 | 112 |  | 0.71 |
| T4 | SEC | Chi-square | 13 | 38 | 76 | 238 | 0.00 | 0.98 |
| T4 | RES | Chi-square | 21 | 69 | 68 | 207 | 0.02 | 0.90 |
| T4 | SED | Chi-square | 67 | 206 | 22 | 70 | 0.00 | 1.00 |
| T4 | RPC | Chi-square | 69 | 209 | 20 | 67 | 0.04 | 0.84 |
| T4 | CDB | Chi-square | 24 | 64 | 65 | 212 | 0.34 | 0.56 |
| T4 | TPI | Fisher | 0 | 8 | 89 | 268 |  | 0.21 |
| T5 | SEC | Chi-square | 11 | 40 | 52 | 262 | 0.46 | 0.50 |
| T5 | RES | Chi-square | 14 | 76 | 49 | 226 | 0.11 | 0.74 |
| T5 | SED | Chi-square | 48 | 225 | 15 | 77 | 0.01 | 0.90 |
| T5 | RPC | Chi-square | 49 | 229 | 14 | 73 | 0.03 | 0.87 |
| T5 | CDB | Chi-square | 14 | 74 | 49 | 228 | 0.05 | 0.82 |
| T5 | TPI | Fisher | 0 | 8 | 63 | 294 |  | 0.36 |
| T6 | SEC | Fisher | 6 | 45 | 25 | 289 |  | 0.41 |
| T6 | RES | Chi-square | 2 | 88 | 29 | 246 | 5.02 | **0.03** |
| T6 | SED | Chi-square | 25 | 248 | 6 | 86 | 0.32 | 0.57 |
| T6 | RPC | Chi-square | 25 | 253 | 6 | 81 | 0.15 | 0.70 |
| T6 | CDB | Chi-square | 8 | 80 | 23 | 254 | 0.00 | 0.99 |
| T6 | TPI | Fisher | 0 | 8 | 31 | 326 |  | 1.00 |
| T7 | SEC | Chi-square | 23 | 28 | 120 | 194 | 0.61 | 0.44 |
| T7 | RES | Chi-square | 33 | 57 | 110 | 165 | 0.19 | 0.66 |
| T7 | SED | Chi-square | 103 | 170 | 40 | 52 | 0.73 | 0.39 |
| T7 | RPC | Chi-square | 105 | 173 | 38 | 49 | 0.74 | 0.39 |
| T7 | CDB | Chi-square | 35 | 53 | 108 | 169 | 0.00 | 1.00 |
| T7 | TPI | Fisher | 2 | 6 | 141 | 216 |  | 0.49 |
| T8 | SEC | Chi-square | 8 | 43 | 44 | 270 | 0.01 | 0.92 |
| T8 | RES | Chi-square | 15 | 75 | 37 | 238 | 0.34 | 0.56 |
| T8 | SED | Chi-square | 43 | 230 | 9 | 83 | 1.55 | 0.21 |
| T8 | RPC | Chi-square | 45 | 233 | 7 | 80 | 2.96 | 0.09 |
| T8 | CDB | Chi-square | 8 | 80 | 44 | 233 | 2.00 | 0.16 |
| T8 | TPI | Fisher | 0 | 8 | 52 | 305 |  | 0.61 |
| T9 | SEC | Chi-square | 15 | 36 | 108 | 206 | 0.29 | 0.59 |
| T9 | RES | Chi-square | 31 | 59 | 92 | 183 | 0.00 | 0.97 |
| T9 | SED | Chi-square | 104 | 169 | 19 | 73 | 8.61 | **< 0.001** |
| T9 | RPC | Chi-square | 104 | 174 | 19 | 68 | 6.51 | **0.01** |
| T9 | CDB | Chi-square | 31 | 57 | 92 | 185 | 0.05 | 0.83 |
| T9 | TPI | Fisher | 3 | 5 | 120 | 237 |  | 1.00 |
| T10 | SEC | Fisher | 0 | 51 | 8 | 306 |  | 0.61 |
| T10 | RES | Fisher | 1 | 89 | 7 | 268 |  | 0.69 |
| T10 | SED | Fisher | 4 | 269 | 4 | 88 |  | 0.11 |
| T10 | RPC | Fisher | 4 | 274 | 4 | 83 |  | 0.10 |
| T10 | CDB | Fisher | 2 | 86 | 6 | 271 |  | 1.00 |
| T10 | TPI | Fisher | 0 | 8 | 8 | 349 |  | 1.00 |
| T11 | SEC | Chi-square | 28 | 23 | 169 | 145 | 0.00 | 1.00 |
| T11 | RES | Chi-square | 52 | 38 | 145 | 130 | 0.51 | 0.48 |
| T11 | SED | Chi-square | 151 | 122 | 46 | 46 | 0.58 | 0.45 |
| T11 | RPC | Chi-square | 153 | 125 | 44 | 43 | 0.37 | 0.55 |
| T11 | CDB | Chi-square | 53 | 35 | 144 | 133 | 1.51 | 0.22 |
| T11 | TPI | Fisher | 5 | 3 | 192 | 165 |  | 0.73 |
| T12 | SEC | Fisher | 1 | 50 | 1 | 313 |  | 0.26 |
| T12 | RES | Fisher | 2 | 88 | 0 | 275 |  | 0.06 |
| T12 | SED | Fisher | 1 | 272 | 1 | 91 |  | 0.44 |
| T12 | RPC | Fisher | 2 | 276 | 0 | 87 |  | 1.00 |
| T12 | CDB | Fisher | 1 | 87 | 1 | 276 |  | 0.43 |
| T12 | TPI | Fisher | 0 | 8 | 2 | 355 |  | 1.00 |
| T13 | SEC | Chi-square | 26 | 25 | 186 | 128 | 0.91 | 0.34 |
| T13 | RES | Chi-square | 56 | 34 | 156 | 119 | 0.63 | 0.43 |
| T13 | SED | Chi-square | 164 | 109 | 48 | 44 | 1.45 | 0.23 |
| T13 | RPC | Chi-square | 167 | 111 | 45 | 42 | 1.57 | 0.21 |
| T13 | CDB | Chi-square | 48 | 40 | 164 | 113 | 0.42 | 0.52 |
| T13 | TPI | Fisher | 5 | 3 | 207 | 150 |  | 1.00 |
| T14 | SEC | Chi-square | 5 | 46 | 37 | 277 | 0.03 | 0.86 |
| T14 | RES | Chi-square | 10 | 80 | 32 | 243 | 0.00 | 1.00 |
| T14 | SED | Chi-square | 31 | 242 | 11 | 81 | 0.00 | 1.00 |
| T14 | RPC | Chi-square | 31 | 247 | 11 | 76 | 0.04 | 0.85 |
| T14 | CDB | Chi-square | 7 | 81 | 35 | 242 | 1.01 | 0.31 |
| T14 | TPI | Fisher | 1 | 7 | 41 | 316 |  | 1.00 |
| T15 | SEC | Fisher | 3 | 48 | 14 | 300 |  | 0.72 |
| T15 | RES | Fisher | 6 | 84 | 11 | 264 |  | 0.39 |
| T15 | SED | Fisher | 12 | 261 | 5 | 87 |  | 0.78 |
| T15 | RPC | Fisher | 12 | 266 | 5 | 82 |  | 0.57 |
| T15 | CDB | Fisher | 4 | 84 | 13 | 264 |  | 1.00 |
| T15 | TPI | Fisher | 0 | 8 | 17 | 340 |  | 1.00 |

## Outcomes of bivariate inferential statistics for continuous predictors

| **Feature** | **Outcome** | **Test** | **Median for Group with Target Variable Present** | **Median for Group with Target Variable Absent** | **W Statistic** | ***p*** |
| --- | --- | --- | --- | --- | --- | --- |
| NOS | SEC | Mann-Whitney U | 2.00 | 2.00 | 9,041.50 | 0.12 |
| NOS | RES | Mann-Whitney U | 2.00 | 2.00 | 11,826.00 | 0.51 |
| NOS | SED | Mann-Whitney U | 2.00 | 2.00 | 13,321.00 | 0.37 |
| NOS | RPC | Mann-Whitney U | 2.00 | 2.00 | 12,646.00 | 0.50 |
| NOS | CDB | Mann-Whitney U | 2.00 | 2.00 | 11,984.00 | 0.81 |
| NOS | TPI | Mann-Whitney U | 2.50 | 2.00 | 1,524.00 | 0.74 |
| PRD | SEC | Mann-Whitney U | 3.00 | 3.00 | 8,904.00 | 0.13 |
| PRD | RES | Mann-Whitney U | 3.00 | 3.00 | 12,464.00 | 0.90 |
| PRD | SED | Mann-Whitney U | 3.00 | 3.00 | 12,036.50 | 0.48 |
| PRD | RPC | Mann-Whitney U | 3.00 | 3.00 | 11,750.00 | 0.63 |
| PRD | CDB | Mann-Whitney U | 3.00 | 3.00 | 12,629.00 | 0.54 |
| PRD | TPI | Mann-Whitney U | 3.00 | 3.00 | 1,489.50 | 0.81 |
